# Preparation, Performance and Challenges of Catalyst Layer for Proton Exchange Membrane Fuel Cell

**DOI:** 10.3390/membranes11110879

**Published:** 2021-11-15

**Authors:** Meng Xie, Tiankuo Chu, Tiantian Wang, Kechuang Wan, Daijun Yang, Bing Li, Pingwen Ming, Cunman Zhang

**Affiliations:** 1 School of Automotive Studies, Tongji University (Jiading Campus), 4800 Cao’an Road, Shanghai 201804, China; xiemeng@tongji.edu.cn (M.X.); 1911679@tongji.edu.cn (T.C.); 1933503@tongji.edu.cn (T.W.); 2011672@tongji.edu.cn (K.W.); yangdaijun@tongji.edu.cn (D.Y.); pwming@tongji.edu.cn (P.M.); zhangcunman@tongji.edu.cn (C.Z.); 2 Clean Energy Automotive Engineering Center, Tongji University (Jiading Campus), 4800 Cao’an Road, Shanghai 201804, China

**Keywords:** proton exchange membrane fuel cell, membrane electrode assembly, catalytic layer, preparation, drying process, degradation

## Abstract

In this paper, the composition, function and structure of the catalyst layer (CL) of a proton exchange membrane fuel cell (PEMFC) are summarized. The hydrogen reduction reaction (HOR) and oxygen reduction reaction (ORR) processes and their mechanisms and the main interfaces of CL (PEM|CL and CL|MPL) are described briefly. The process of mass transfer (hydrogen, oxygen and water), proton and electron transfer in MEA are described in detail, including their influencing factors. The failure mechanism of CL (Pt particles, CL crack, CL flooding, etc.) and the degradation mechanism of the main components in CL are studied. On the basis of the existing problems, a structure optimization strategy for a high-performance CL is proposed. The commonly used preparation processes of CL are introduced. Based on the classical drying theory, the drying process of a wet CL is explained. Finally, the research direction and future challenges of CL are pointed out, hoping to provide a new perspective for the design and selection of CL materials and preparation equipment.

## 1. Introduction

**Figure 1:**
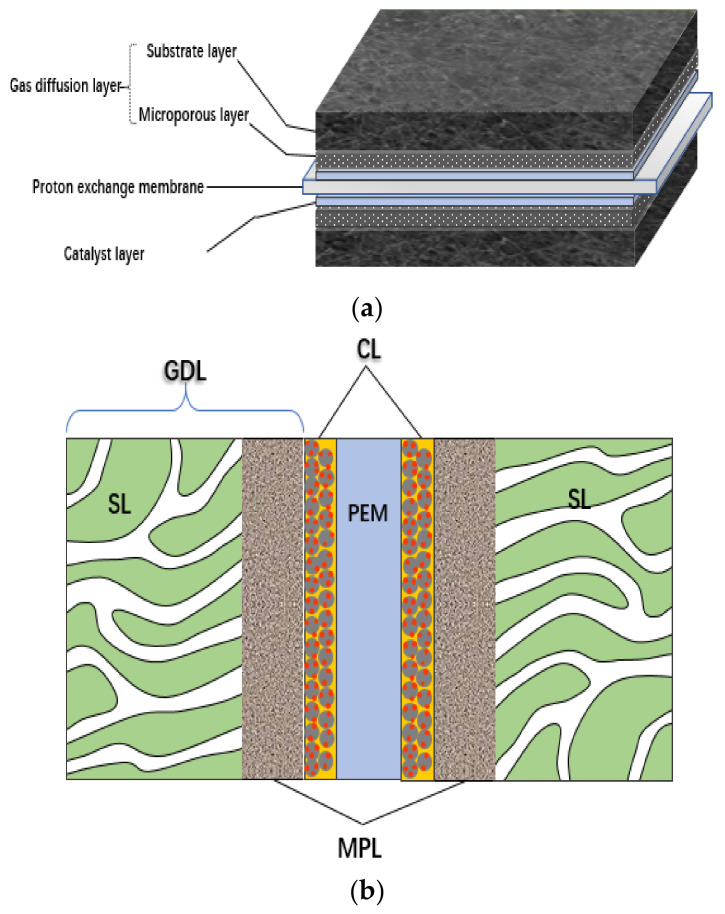
(**a**) Schematic diagram of MEA structure and composition. (**b**) Schematic diagram of MEA cross-section.

At present, the cost and durability of a fuel cell are the key factors that hinder its large-scale commercial application [1,2,3], which is closely related to the properties of membrane electrode assembly (MEA). MEA is the place where electrochemical reaction takes place in the fuel cell, and it is the key component to convert chemical energy into electrical energy. It consists of a proton exchange membrane (PEM), a gas diffusion layer (GDL) (including the substrate layer (SL) and microporous layer (MPL)), a catalyst layer (CL), a sealing frame and other parts [4]; the corresponding schematic diagram of the structure is shown in Figure 1.

During the power generation process of a fuel cell stack, the properties of MEA affect the transportation of reaction gas, protons and electrons; formation of three phase boundary; output of heat; and production of water [5]. The performance and durability of MEA under actual operating conditions largely determine the overall power density and operating lifetime of the stack [6]. The proton conductivity of PEM, the content and distribution of ionomer in CL, and the characteristics of the proton conduction determine the proton transfer ability of MEA. The electron transfer efficiency of GDL and CL determines the electron transfer ability in MEA. The porosity, pore distribution and hydrophilic/hydrophobic properties of CL and GDL directly affect the diffusion of reactant gas and the discharge of product water [7]. After a long time of operation, the interface structure and properties of PEM|CL, CL|MPL and MPL|SL may change gradually. At the same time, the degradation of PEM, agglomeration or loss of platinum (Pt) particles, degradation of ionomer, and corrosion of carbon support may occur in MEA, which will affect the power generation performance and durability of the fuel cell.

According to the U.S. Department of Energy (DOE) 2020 fuel cell technical specifications, under the specified environment and durability test conditions, the power density of a fuel cell stack should be more than 1000 mW cm^−2^ at the rated power point, and the decay should be less than 10% at the rated power point after 4000 h of operation [8]. In the actual operation process of the stack, each functional layer matches and coordinates with each other, which jointly guarantee the mass transfer, catalysis, conduction and other capabilities of each functional layer and jointly affect the performance and durability of the stack. Due to the Buckets effect, the durability of any component of fuel cell stack, such as the bipolar plate, MEA and sealing components, affect the durability of the stack. If only the influence of MEA is considered, the thermal stability, chemical stability and mechanical properties of its components (PEM, CL and GDL) have a great influence on the durability of the stack [9,10,11].

As mentioned above, CL is the place where the electrochemical reactions take place, is the core component of PEMFC. Its composition, proportion and structure affect the proton, electron transfer, heat and mass transport. Therefore, it has always been the focus of scientific research. Within the CL, the reaction gas must have enough space (pores) to transfer; the protons and electrons must have the conduction medium; this is the most basic requirement of CL. Therefore, firstly, CL should have porous properties to achieve rapid transfer of hydrogen and air, reduce the mass transfer loss and remove the excessive water generated in the reaction process simultaneously. Secondly, the transfer of electrons requires CL to have good conductivity; the smaller the electric resistance, the smaller the ohmic loss. Thirdly, the contact between CL and ionic liquids should be close, so that the protons produced by the reaction can be smoothly transferred from anode to cathode. Finally, CL should be thin enough, its thickness not only affects the mass and charge transfer velocity but also relates to the utilization efficiency of the catalyst.

The typical CL is composed of carbon supported Pt catalyst and ionomer [12,13], so its degradation includes the degradation of catalyst active particles, carbon support and Nafion. The degradation of Pt particles mainly includes the Ostwald ripening effect, migration, agglomeration and loss of Pt particles. The increase of Pt particles size or loss of Pt particles result in the reduction of ECSA and catalytic efficiency [14]. The dissolved Pt ions may even enter the ionomer of PEM or CL, affecting the proton conductivity [15]. Carbon corrosion refers to the oxidation of carbon support into CO_2_ or CO under high potential in a fuel cell environment, which makes the Pt particles originally loaded on carbon support fall off. On the one hand, the fallen Pt particles lose their catalytic activity due to the lack of electron transport channel [16]; on the other hand, the loss of support also causes the collapse of CL structure, increasing the transmission resistance of water and gas. Nafion plays the role of both binder and proton conductor at the same time in MEA, and its degradation also causes the collapse of CL structure, thus changing the pore composition and structure of the original CL. Moreover, the destruction of the Nafion binding network inevitably affects the proton transfer efficiency in MEA. The decay mechanism of these materials in CL is described in detail in Section 2.5.4.

**Figure 2:**
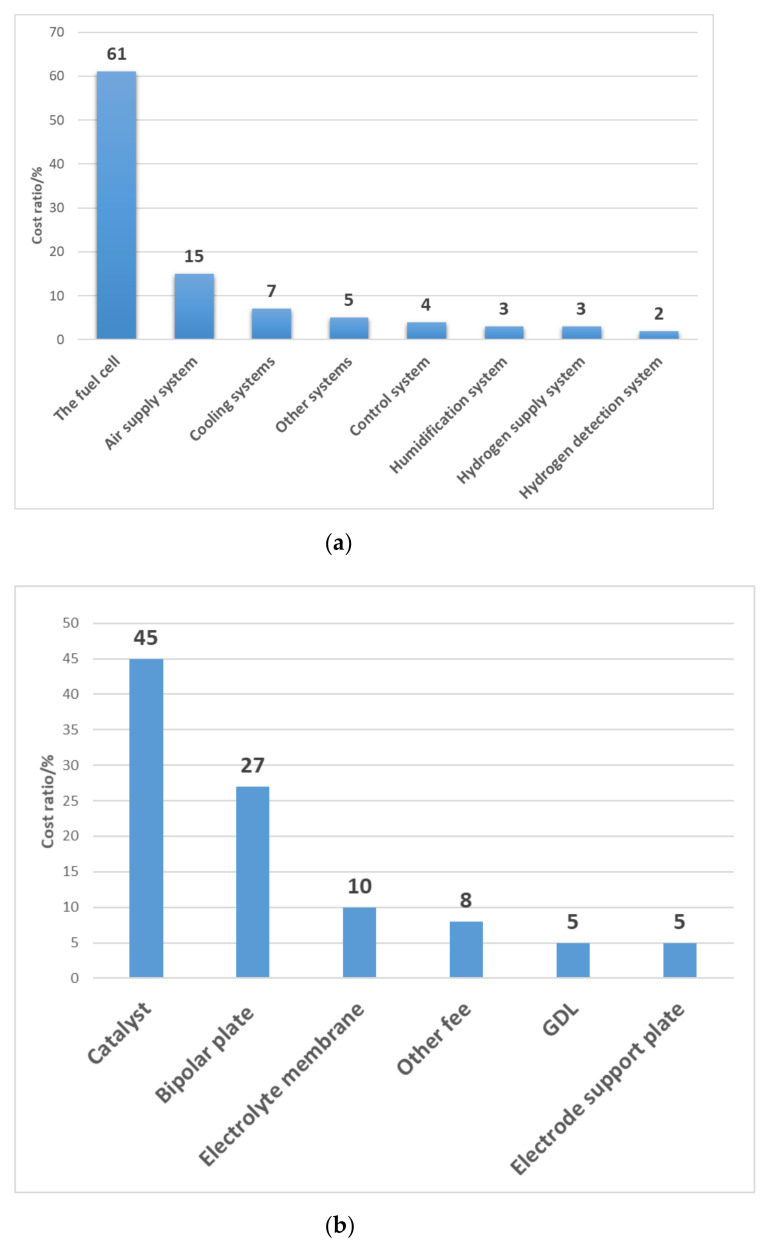
Cost structure of fuel cell system (**a**) and stack (**b**) [17] (Copyright 2015 DOE).

According to the statistical results of U.S. DOE, the fuel cell stack accounts for the largest proportion of the cost of a fuel cell system, as shown in Figure 2a, which is close to 60%. The cost of each component of the stack is shown in Figure 2b [17]. As the key material in CL, catalyst accounts for about 45% of the fuel cell stack cost.

From the cost structure in Figure 2, it can be seen that the method to reduce the cost of stack still needs to start with the improvement of basic materials. Due to the need for noble metals such as Pt, the catalyst accounts for the largest proportion of each component of the fuel cell stack. Developing catalysts with good stability and low cost, while ensuring their catalytic performance is not reduced, is the goal pursued by current studies [18]. At present, the fuel cell is still in the stage of small-scale demonstration application. In order to continue to expand its commercial demonstration application, it is necessary to continuously develop and improve the material and structure of key components and make breakthroughs in performance, durability and cost. CL is the most important part of this process. If MEA is the heart of fuel cell stack, so CL is the heart of MEA: the decay of CL leads to a decrease in catalytic activity, which will directly affect the performance of MEA, and the catalyst in CL accounts for nearly half of the cost of stack. Therefore, the following contents mainly focuses on CL.

## 2. Composition and Structure of CL

### 2.1. Composition Materials of CL and Their Functions

**Figure 3:**
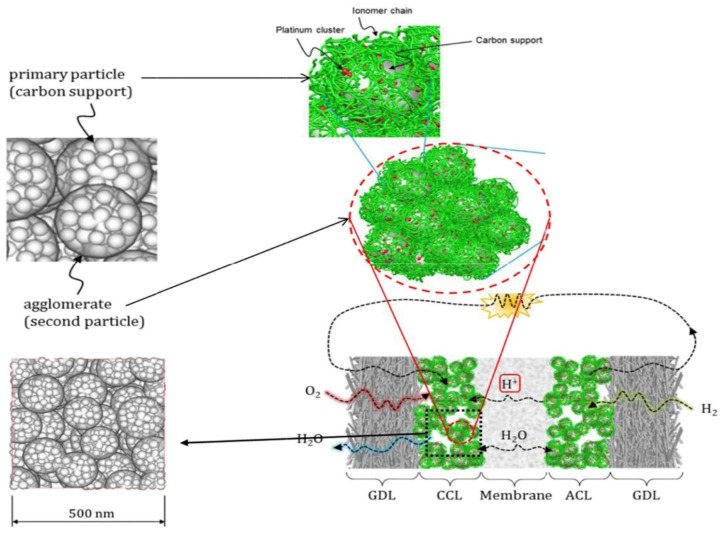
Schematic diagram of CL composition [21]. Copyright 2017 Elsevier.

The construction of the CL is mainly considered in regard to two aspects: one is to improve the catalyst activity as much as possible; the other is to design a reasonable structure of CL to create as many three-phase interfaces as possible [19,20]. However, most fundamentally, the above two aspects are inseparable from the material properties of CL itself. As mentioned above, and as shown in Figure 3, the typical CL materials are mainly ionomer and catalyst nanoparticles.

Ionomer: In the early stage of fuel cell development, PTFE was mainly used as the binder material in CL; the utilization rate of Pt particles was very low, and the Pt loading was generally more than 4 mg cm^−2^ [22]. At present, DuPont’s Nafion resin has been widely used in fuel cells as the ionic liquid. We know that the ionomer (ionic liquids) in CL acts both as binder and proton conductor [23], when the Nafion content is low, the proton transport ability in CL is weak, and the binding strength of CL is also insufficient. When the Nafion content is high, the proton transport capacity and bonding strength of CL will be enhanced correspondingly, but due to the electrical insulation of Nafion itself, the electron transport in CL will be affected [24]. At the same time, the higher ionic liquids content will also reduce the porosity in CL, affecting the diffusion of reaction gas and the timely discharge of water [25]. Therefore, the amount of ionic liquids in CL should be just right to maintain the high-performance output capacity of CL [26]. Generally, the mass ratio of Nafion to carbon support is used to describe the content of Nafion in CL, i.e., I/C ratio [27] (ionomer/carbon support). The value of I/C ratio not only affects the dispersion of slurry but also has great influence on the morphology (crack) and properties of CL. The optimal I/C ratio is related to the platinum loading and is also closely related to the type and properties of the used ionomer and carbon support, and the anode and cathode are also different, with common values ranging from 0.2 to 1.5.

Catalysts: The structure, morphology and surface characteristics (hydrophilic and hydrophobic characteristics, etc.) of the catalyst will affect the interaction between dispersant and ionomer, thus affecting the structure and morphology of CL. According to the composition of materials, catalysts can be divided into Pt based catalysts, non-precious metal catalysts and non-metal catalysts. Due to the scarcity and high cost of Pt, low-Pt and even Pt-free catalysts have become the hotspots of scientific research, but at present, the application of catalysts is still mainly focused on Pt based catalysts [28,29]. Typical commercial catalysts are carbon supported Pt catalysts or carbon supported Pt alloy catalysts produced by Tanaka in Japan and Johnson Matthey in the UK, with a Pt content ranging from 10 to 70%, Such as JM-Hispec 4000 (40%), and JM-Hispec 9100 (60%), which are commonly used catalyst types. The carbon binding network formed by the support materials in the catalyst constitutes the internal electron transport channel of MEA, and the Pt particles carried on the support not only provide the electron transport channel for hydrogen oxidation reaction (HOR)/oxygen reduction reaction/(ORR) but also provide the activation site for the catalytic reaction. The junction of reaction gas, catalyst and ionomer is usually called three-phase boundary [30,31,32,33]. The three-phase boundary is the location of redox reaction in CL, which can essentially be regarded as the intersection of electron, proton and molecular (reactant gas) channels. Of course, the larger the three-phase boundary, the more efficient the redox reaction will be. At present, different methods have been used to reduce Pt loading and improve the activity and durability of catalysts, including adjusting the structure and morphology of Pt particles [34,35,36], adjusting the exposure of high active crystal surface of Pt particles [37,38], constructing alloying system [39,40,41,42,43,44,45], developing a non-Pt based catalyst [28,29], improving the properties of carbon support [46,47,48], replacing carbon supports such as Ti [49,50] and silica [51], and recovering precious metals from the catalyst in the post-treatment stage [52].

U.S. DOE has clear testing methods and standards for catalyst performance degradation tests [53]: the 25–50 cm^2^ single cell was scanned by square wave potential cycle in-situ, and the temperature was kept at 80 °C, with 200 mL min^−1^ hydrogen for the anode and 75 mL min^−1^ for the cathode. The humidity on both sides was kept at 100%, and the pressure was kept at atmospheric. The scanning potential was maintained for 3 s at 0.6 V and 3 s at 0.95 V, and a total of 30 k cycles were performed. After a certain number of cycles is completed, a polarization curve test is carried out to obtain the change of MEA performance. The target is that, after 30 k cycles, the decay rate of catalyst mass specific activity should be less than 40%; the polarization curve voltage degradation should be less than 30 mV@0.8 A cm^−2^, and the decay rate of ECSA should be less than 40%.

With the development of science and technology, the interaction between the ionic liquid (ionomer) and catalyst has been also a focus of research. The ionomer consists of a hydrophobic polytetrafluoroethylene main-chain and a perfluoroether side chain with a hydrophilic sulfonic acid group at the end. Subbaraman [54,55] et al., found that the sulfonic groups of the side chain were adsorbed on the surface of Pt nanoparticles, resulting in a decrease in the active area of the catalyst and a decrease in the ORR kinetics. This result was also proved by Shinozaki [56] and Kodama et al. [57,58]. The study by Kodama [59] et al. also showed that reducing the side chain length of the ionomer was conducive to reducing the toxic effect of Nafion side chain sulfonic acid groups on ORR, possibly because the flexibility of the side chain was reduced.

In addition, CL is generally prepared on PEM, and located in the center of MEA. Its role is to separate the reaction between anode and cathode, separate the gas and the electrons produced by the reaction, and it also needs to act as the channel for proton transport from the anode to cathode [60]. According to its function and working environment, the following requirements are proposed for PEM [61,62]:

(1) Proton conductivity. The higher the proton conductivity, the smaller the proton conductivity resistance of PEM, which makes the overall internal resistance of MEA decrease, and the current density increase. Because protons transports in the form of hydronium ions, the proton conductivity is usually related to the water content and temperature of PEM, so it is often necessary to test at a specific temperature and humidity when evaluating their proton conductivity [63,64].
(2) Gas permeation rate. One of the most important functions of PEM is to separate the reactions on both sides of MEA, so it is necessary to have a low gas permeability to prevent the fuel from mixing with the oxides, otherwise it will lead to local overheating, which greatly affects the efficiency and lifetime of fuel cell.
(3) Dry-wet conversion characteristics. PEM water content not only affects the proton conductivity but also its mass and volume, and the humidity and temperature of the electrode are not constant during operation. The ideal PEM requires higher water content at lower humidity or higher temperature, to prevent the PEM from drying and affecting its performance. However, when the water content of PEM is too high, its mass and volume will increase accordingly. The change of mass is usually expressed by the water absorption rate, and the change of volume is expressed by the swelling rate. The smaller the swelling rate, the better because in the process of CL fabrication, excessive swelling rate will affect the degree of binding between CL and PEM, which may lead to interfacial delamination in severe cases and increase the proton conduction resistance, thus affecting the MEA performance.
(4) Stability. The PEM for fuel cell requires good chemical stability, thermal stability, and mechanical stability. Sufficient stability ensures the durability of PEM and thus prolong its lifetime. PEM operates under acidic conditions (take PEMFCs for example), and its temperature varies during the operating. Under such operating conditions, it must have good acid resistance. Thermal stability ensures that the PEM does not degrade at the working temperature. Higher mechanical strength prevents PEM damage during assembly.

Based on the above performance requirements, the most widely used PEM currently is the Nafion membrane developed and produced by the DuPond(E.I. du Pont de Nemours and Company, Fayetteville, NC, USA) company in the US, such as Nafion^®^ N-115, N-117, NER-211.

### 2.2. Structural Characteristics of CL

**Figure 4:**
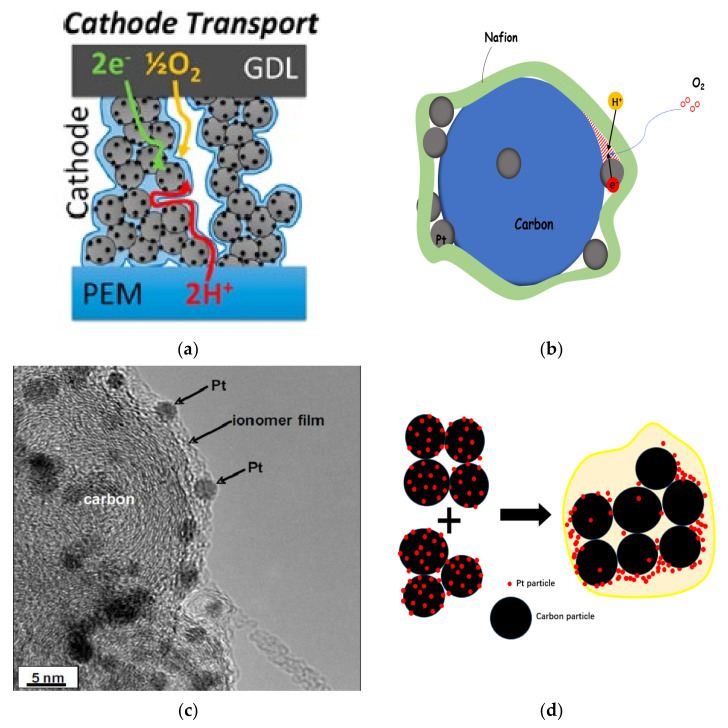
(**a**) Schematic diagram of MEA cathode side structure and electron and proton [67]. Copyright 2015 American Chemical Society. (**b**) Schematic diagram of three-phase boundary of PEMFC cathode. (**c**) Microstructure of Pt particle/ionomer/carbon support as shown by scanning electron microscope (SEM) [68]. Copyright 2006 ECS transactions. (**d**) Secondary distribution of Pt nanoparticles on support during the CL preparation.

The material composition, proportion and pore structure of CL have a great influence on its properties [65]. According to the working principle of the fuel cell, transmission channels for electrons, protons, reaction gas and product water must be provided. In fact, CL is composed of particles of different sizes (primary particles and secondary aggregates) and pores (primary pores and macropores). During the operation of a fuel cell, the diffusion of reaction gas, HOR and ORR, generation and transmission of hydrogen protons and electrons, generation and transfer of water and other processes occur simultaneously, which are closely related to the heterogeneous pore structure of CL [66], as shown in Figure 4a.

The catalyst particles and Nafion are dispersed by solvents (such as ethanol, n-propanol, etc.), and after coating and drying, a disordered distribution of porous structure is formed in CL [69]. The dry CL thickness ranges from 10 to 20 μm. Generally, the diameter of carbon support particles is 30~50 nm, and that of Pt particles is 2~5 nm. In most cases, carbon particles will form carbon aggregates under the bonding action of Nafion, and many Pt particles are distributed on the surface. The aggregate formed by a single carbon particle loaded with multiple Pt nanoparticles is called primary particle, while the aggregate formed by carbon aggregate and many Pt particles is called secondary particle (or secondary aggregate), as shown in Figure 3 [21]. The diameter of secondary particles is between 100~300 nm, and there are a lot of internal pores with a diameter of about 1~20 nm, which are usually inaccessible to the ionomer, and they are called primary pores. The gaps between secondary particles are generally much larger than 20 nm, which are called macropores [70,71]. Figure 4b–d, respectively, illustrate the composition of the three-phase boundary, the actual microstructure of the three-phase boundary and the secondary redistribution of Pt nanoparticles on the support during the preparation of CL.

### 2.3. Interface of CL

**Figure 5:**
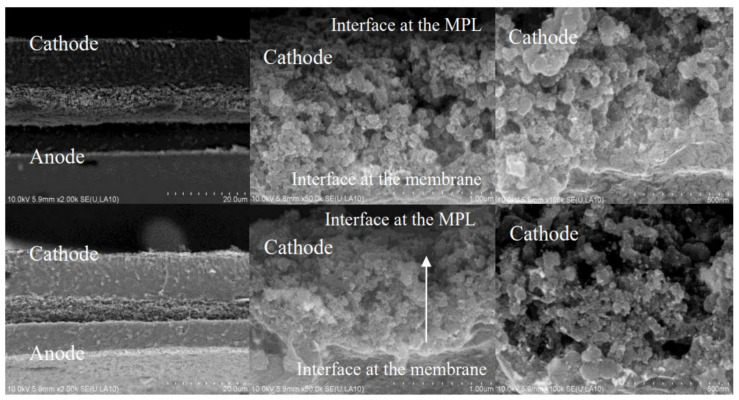
The SEM images of the cross section of MEA before (first row) and after (second row) 30 k cycles [72]. Copyright 2017 ECS transactions.

CL is coated on PEM and connected to the MPL of GDL, so there are two main interfaces, one is the PEM|CL interface, and the other is the MPL|CL interface, as shown in Figure 5. The complexity is that the interface will change with the working conditions, resulting in a change of MEA structure, causing irreversible deterioration of MEA performance.

#### 2.3.1. Interface of PEM|CL

It is well known that the degree of contact between PEM and CL affects the electrochemical performance of MEA, because it is closely related to the impedance of proton transfer. Most of the existing CL fabricate processes use Nafion as binder to reduce the proton transmission resistance and improve the cell performance [73]. Compared with the traditional gas diffusion electrode (GDE) process or transfer printing method, part of the catalyst slurry in the electrode prepared by catalyst coated membrane (CCM) process, will penetrate into the PEM, reducing the proton transfer resistance and water transfer resistance [25]. Compared with the GDE process, the most obvious change of CCM process is that it improves the interface characteristics of PEM and CL, improves the effective utilization rate of Pt and, finally, improves the power density of MEA [74]. However, both of these processes are based on the two-dimensional flat membranes. In recent years, many new and improved technologies have tried to replace the commonly used flat membrane but forming various patterns on the membrane started to be considered from a three-dimensional perspective with the interface characteristics of PEM|CL [75,76,77,78,79,80].

Bae et al. [77] used a linear type of Nafion membrane to compare with a planar membrane (Pt loading 0.2 mg cm^−2^, 5 cm^2^, N-212, thickness 50 μm). They found that the cell power was greatly improved after the membrane was replaced, especially when the voltage was lower than 0.6 V (i.e., the concentration polarization region). In three different experiments, MP2 type (2 μm membrane) is the best. Compared with planar membrane under the same conditions, although the ECSA reduced by 2%, the high frequency resistance (HFR) decreased by 22.3%, the low frequency resistance (LFR) decreased by 7.3%, and the maximum power reached 0.70 W cm^−2^, which was 25% higher than that of the planar membrane (0.56 W cm^−2^). The utilization rate of cathode Pt reached 3.5 kW g_pt_^−1^.

Kim et al. [78] printed a tapered pattern on the membrane (Pt loading 0.12 mg cm^−2^, N-212, thickness 50 μm). When the current density is greater than 0.8 A cm^−2^, the improvement of water management by tapered membrane is equivalent to a 50% increase in power density. They mainly believe that this kind of tapered membrane has three advantages: Firstly, the local membrane is thin, resulting in a relatively low impedance. Secondly, this structure increases the contact area between CL and PEM. Thirdly, this structure enables optimizing the water management within the electrode.

Cho et al. [79] proposed a Lego-like mold structure composed of cylinders with different diameters in the microscopic scale. Compared with the flat membrane, the ECSA of the electrode prepared by the N212 membrane is increased from 58.11 to 69.12 m^2^ g^−1^. The power density increases by 42.3% at 100 kPa and only by 10% at 150 kPa. The authors believe that the decrease of the membrane resistance and the increase of the ECSA lead to the improvement of the performance.

Zhang et al. designed a novel dual CL structure in the PEMFC cathode, the inner CL (close to the gas diffusion layer) with low-Pt-loading, and the outer CL (next to the membrane) with high-Pt-loading. The experimental results show that the performance of GDE prepared by double CL is significantly higher than that of GDE with traditional single CL. It is attributed to the intimate contact between CL and membrane, an increased Pt utilization and the reduced mass transfer limitations [81].

#### 2.3.2. Interface of CL|MPL

The contact degree, hydrophilicity and pore structure of the CL|MPL interface are important design parameters of MEA. This interface mainly affects the electron transfer impedance within the MEA, as well as the distribution of reactant gas and the discharge of product water [82]. In MPL, only the pores which are connected with each other and extend to the surface of CL can effectively transmit the reaction gas. In terms of electrode performance, the CL|MPL interface features are closely related to ohmic loss and mass transfer loss. Schneider et al. [83] showed that MPL could significantly increase the contact between CL and GDL and reduce the contact resistance of the interface. Kannan et al. [84] prepared MPL by themselves, coated PTFE and carbon powder slurry in a gradient distribution. The outermost layer was coated with a thin hydrophilic layer (adjacent to CL). They found that MEA prepared by this process has good water retention, especially under the test conditions of high temperature and low humidity. Chen et al. [85] added another MPL on the basis of the original one, which was composed of carbon powder and PTFE, and was sintered on the original MPL. Compared with the original MPL, the porosity and pore size of the new ones are different, and the pore structure presents a gradient distribution, which improves the water management level of MEA. Li et al. [86] used six different types of carbon black to prepare a cathode MPL and found that the performance of single cell prepared by ACET and Vulcan XC-72 was similar. The author believes that the reasonable pore characters could remove the water in the cathode effectively and improve the performance of the single cell. When P(VDF-HFP) with different content is replaced by PTFE as binder, the GDL surface resistance prepared by different mass fraction P(VDF-HFP) (10%,20%,30%,40%) was greater than that of GDL prepared by 30% PTFE, so the performance of a single cell is not as good as that of 30% PTFE as binder.

### 2.4. Transmission, Failure and Optimization in CL

General situation of CL internal transport: The electron transport channel is a carbon binding network composed of carbon support particles. The proton transport channel consists of an ionomer network (proton conductor). At the same time, the two materials combine with each other, and the proton conductor serves as the binder, forming a complex three-dimensional porous network structure, which is used to transfer the reaction gas and product water [87].

**Figure 6:**
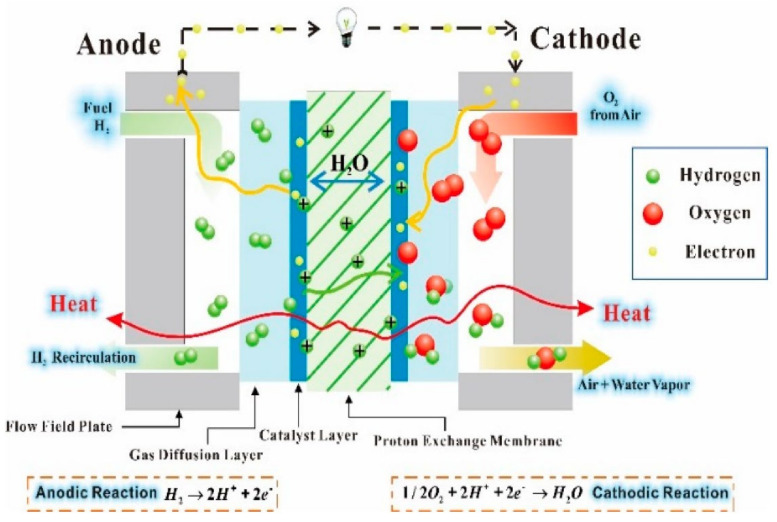
Schematic of a PEMFC [92]. Copyright ©2019 ChemEngineering.

Taking PEMFC as an example, as shown in Figure 6, HOR occurs when hydrogen enters the anode, which is a reversible electrode reaction with the exchange current density (Pt electrode) of 10^−3^ A cm^−2^. ORR, on the other hand, is a highly irreversible electrode reaction, and its exchange current density (Pt electrode) is 10^−9^ A cm^−2^ or even lower [88,89,90]. Therefore, ORR is the control step of fuel cell reaction [91], and its reaction rate determines the overall electrochemical reaction rate of the fuel cell.

The three-phase boundaries (TPBs) are located in the porous structure of CL. The formation of the porous structure is mainly due to the interaction of dispersed solvent, ionomer and catalyst particles during evaporation. Therefore, the formation of three-phase boundary is closely related to the type, properties and quantity of solvent in the slurry, material properties of catalyst and ionomer, dispersion method of slurry, coating and drying process. It is a multi-factor coupling process from dynamic to static. Due to it being difficult to control the slurry preparation, dispersion, coating and drying process of the CL accurately, the pore structure in CL is random and disordered, and some catalysts and ionomers cannot reach the conditions of three-phase reaction active sites, that is, these catalysts and ionomers will inevitably be wasted, leading to a significant reduction in catalyst utilization rate. Here are some common situations:

(1) The porous structure of carbon support allows Pt particles to enter, but the ionomer cannot reach the corresponding site, and Pt particles lack proton transfer channel and reduce their activity. In general, micropores smaller than 20 nm prevent the entry of ionomer.
(2) When Pt particles enter the pores of carbon support, the ionomer may block the entrance of pores, increase the diffusion resistance of reaction gas, and reduce the catalytic efficiency of Pt particles.
(3) If the ionomer is too thick, since the gas need be transferred to the three-phase boundary through the ionomer, the gas concentration will gradually decrease from the outside to the inside, forming in a concentration gradient, resulting in the reduction of catalytic efficiency of Pt particles.
(4) During the preparation of catalyst and slurry or the coating of CL, the catalyst particles themselves will agglomerate. All of these will lead to a decrease in the utilization rate of Pt particles in CL (as shown in Figure 4d).

Middleman [93] proposed an ideal CL model, in which Pt atoms are evenly distributed around electronic conductors such as carbon rods, and then covered with proton conductor-Nafion with appropriate thickness. The gap between carbon rods allows the reactant gas to enter and remove product water. The most important feature of ideal CL structure is the establishment of the orderly transmission channels of electrons, protons, reaction gas and product so that there are enough three-phase reaction boundaries, and the resistance of proton, electron transfer, gas and water transmission is small enough so as to improve the utilization rate of Pt particles [94].

The proton, electron transfer, gas transmission and water discharge processes within the CL of PEMFC are described below. Anode fuel gas, i.e., hydrogen, enters MEA through the flow field distribution of a bipolar plate. Relying on the internal porous structure of GDL and CL, the second fine distribution of hydrogen reaches the active site of HOR. The uniformity of gas distribution in these processes is greatly related to the consistency of a fuel cell stack. The uniformity of gas distribution mainly depends on the porosity and pore size distribution of GDL and CL.

Under the action of the fuel cell anode catalyst, the following HOR occurs:

$$2H_{2}\to 4H^{+}+4e^{-}\;E0=0.000\;V\;\mathrm{vs}.\;\mathrm{RHE}$$

The anode gas reaches the active point through the pores of CL, makes contact with Pt particles, and is adsorbed on the surface of Pt atoms. The bond between hydrogen molecule and Pt atoms leads to the decrease of H-H bond energy until it disappears. At this time, each H atom shares an electron with the Pt atom. With the help of the random movement of water molecules, hydrogen protons leave the surface of Pt atoms in the form of hydrated hydrogen ions (H_3_O^+^). Driven by the concentration gradient, H_3_O^+^ is transferred in the proton conductor. Specifically, the sulfonate acid side chain in ionomer will form a cluster structure due to hydration, and H_3_O^+^ in the cluster can realize the transfer from anode to cathode depending on the concentration difference. PEM and Nafion in CL constitute the hydrogen proton transfer pathway from anode to cathode. At the same time, when H_3_O^+^ detaches from the surface of Pt atoms, the generated electrons are transferred to the cathode through Pt particles, carbon particle binding network, carbon fiber inside GDL, external circuit, etc.

Similarly, the reactant gas of fuel cell cathode is usually pure oxygen or air (oxygen volume fraction 21%), which is initially distributed through the gas pipeline and bipolar plate flow field and then through the secondary distribution of GDL and CL porous structure of cathode and permeates to the reaction active point in CL.

ORR occurs under the action of fuel cell cathode catalyst, and the equation is as follows:

$$O_{2}+4H^{+}+4e^{-}\to 2H_{2}O\;E0=+1.229\;V\;\mathrm{vs}.\;\mathrm{RHE}\;\left(4e^{-}\mathrm{pathway}\right)$$

$$O_{2}+2H^{+}+2e^{-}\to H_{2}O_{2}\;E0=+0.682\;V\;\mathrm{vs}.\;\mathrm{RHE}\;\left(2e^{-}\mathrm{pathway}\right)$$

$$H_{2}O_{2}+2H^{+}+2e^{-}\to 2H_{2}O$$

There are many explanations for the reaction mechanism of ORR, the most important of which are the two-electron pathway explanation and four-electron pathway explanation. In the two-electron pathway explanation, oxygen molecules are first adsorbed on the surface of Pt atoms. The adsorbed oxygen molecules combine with two hydrogen protons to form HOOH, an intermediate in the adsorbed state, before the O-O bond breaks. H_2_O_2_ is unstable, and it may be further reduced to H_2_O, re-oxidized to oxygen, or detached from the electrode surface in the form of H_2_O_2_. This process is often referred to as the two electron H_2_O_2_ mechanism. There are two possible ways of four electron pathway, which are oxygen dissociation pathway and oxygen binding pathway [95]. The O-O bond adsorbed on the surface of Pt atom breaks directly, and two oxygen atoms are formed in the adsorbed state. It combines with one hydrogen proton to form OH and then combines with another hydrogen proton to form H_2_O. This process is called oxygen dissociation pathway. The oxygen molecule adsorbed on the surface of Pt atom first bonds with a hydrogen proton to form the adsorbed intermediate OOH, then the O-O bond breaks to form OH, which is further reduced to H_2_O, another oxygen atom is reduced to water in the same way. This process is called the oxygen binding pathway. As described above, the reaction equations of two-electron pathway and four-electron pathway are as follows:

2$e^{-}$ pathway:

$$O_{2}{+}^{*}\to O_{2}{}^{*}$$

$$O^{*}+H^{+}\to {\mathrm{OOH}}^{*}$$

$${\mathrm{OOH}}^{*}+H^{+}\to H_{2}O_{2}{}^{*}$$

$$H_{2}O_{2}{}^{*}{+}^{*}\to 2{\mathrm{OH}}^{*}$$

$$2{\mathrm{OH}}^{*}+2H^{+}\to 2H_{2}O$$

4$e^{-}$ oxygen dissociation pathway:

$$O_{2}{+}^{*}\to O_{2}{}^{*}\to 2O^{*}$$

$$2O^{*}+4H^{+}+2e^{-}\to 2{\mathrm{OH}}^{*}$$

$$2{\mathrm{OH}}^{*}+2H^{+}+2e^{-}\to 2H_{2}O$$

4$e^{-}$ oxygen binding pathway:

$$O_{2}{+}^{*}\to O_{2}{}^{*}$$

$$O_{2}{}^{*}+H^{+}\to {\mathrm{OOH}}^{*}$$

$${\mathrm{OOH}}^{*}{+}^{*}+e^{-}\to O^{*}+{\mathrm{OH}}^{*}$$

$$O_{2}{}^{*}+H^{+}+e^{-}\to {\mathrm{OH}}^{*}$$

$$2{\mathrm{OH}}^{*}+2H^{+}+2e^{-}\to 2H_{2}O$$

During the operation of the fuel cell, liquid water is gradually generated on the cathode side, and the rate of water generation is determined by the ORR reaction rate. This part of water plus the water dragged from anode to cathode by electroosmosis minus the water which moves in reverse osmosis to the anode is the amount of residual water on cathode side.

### 2.5. Failure Mechanism of CL

#### 2.5.1. Failure of Pt Particles in CL

As mentioned above in Section 2.4, there are always such Pt particles in CL, otherwise they cannot make contact with the ionomer, or they cannot realize effective gas transmission due to the thickness of the ionomer, or they cannot establish proton and electron transmission channels due to the separate Pt particles separated from carbon support and lose catalytic effect. The isolated Pt particles often come from the corrosion or loss of carbon support during the preparation of the catalyst slurry, such as when there is intense ultrasonic impact or long-time stirring; or from the extreme operating conditions, such as continuous high potential or frequent start and stop conditions. More et al. [68] found that in the case of high I/C ratio, more Pt particles were found around the secondary pores. It is speculated that Pt particles tend to aggregate during slurry preparation. Due to the pore-formation during the drying process, these Pt particles form this arrangement around the pores. Gasteiger et al. [96] found that during the CL formation, Pt particles on the carbon support would shift in position. Before the addition of solvent, Pt particles on the carbon support were generally uniformly distributed. Due to the addition of solvent and ionomer, catalyst particles are covered by the ionomer, which will redistribute Pt particles on the surface of the secondary aggregate, resulting in uneven distribution of Pt particles in the slurry and easy aggregation, reducing the utilization efficiency of the catalyst, as shown in Figure 4d.

#### 2.5.2. Crack of CL

In recent years, the cracking of CL has gradually become a new research hotspot. During the preparation of CL, due to different surface stress and internal drying rate, cracks may appear on the surface of CL [97,98]. Different drying processes often lead to cracking of CL after catalytic coating on PEM. More and more reports have been published on the reasons for the formation of cracks in CL, which may be related to the solvent type and characteristics of catalyst slurry [99,100], the mixing time of catalyst slurry [101], the different amount of ionomer and the properties of coating substrate [102]. In general, the formation of cracks is related to the interaction between catalyst particles and solvent in slurry, as well as the interaction between catalyst particles and ionomer [103]. Cracks in CL must have an impact on the performance and lifetime of fuel cell, and the impact of the results have positive and negative views [104].

On the one hand, Ahn et al. [105] coated catalyst slurry on the specific PEM and stretched MEA by mechanical force, controlled the degree of deformation, changed the crack spacing and type in CL, and studied its influence on the MEA performance. The results show that when the spacing is 20 μm and the strain value is 0.5, the MEA performance prepared by this method is 18% higher than that by the traditional method. Subsequent electrochemical impedance spectroscopy (EIS) results show that the formation of controllable crack can effectively enhance the water transport capacity of the cathode side. Kim et al. [106,107] also conducted a similar study.

On the other hand, Hoffmann et al. [27] studied the occurrence of cracks from the perspective of slurry formulation. The effects of I/C ratio and different types of carbon black on the properties of slurry suspended particles and CL were studied. The results show that the dendritic carbon black has a loose structure, large pore size, small capillary force and few cracks in the CL. The less branched carbon supports are more tightly packed, and the larger capillary force caused by the smaller pores will lead to more cracks during the CL drying process. According to the transfer mechanism of gas, electron and proton in CL, it is very important to avoid cracks during drying because these cracks may lead to gas bypass flow and destroy the proton and electron transport channels, affecting the performance of CL. In addition, CL cracks may be the main cause of PEM cracks or pinholes after the stack operation for a long-term [98,108,109,110,111,112]. Most studies show that the CL cracking seriously affects the MEA lifetime [97,98,108,109,110,111,112,113,114]. These results show that water flooding firstly occurred at the CL crack site and eventually resulted in PEM cracks or pinholes, which was confirmed by X-ray computed tomography (CT) results. This proves that PEM cracks firstly appear at the corresponding CL cracks, leading to the leakage of gas between anode and cathode, which seriously affects the MEA performance and durability.

#### 2.5.3. Flooding of CL

As we know from the previous analysis, proton transfer in the fuel cell is realized in the form of H_3_O^+^, which requires PEM to keep enough wetting. However, the excess water will occupy the gap in CL and hinder the reaction gas to reach the active point, which will affect the high-density output performance of the fuel cell (concentration polarization effect). In severe cases, it may even lead to oxygen starvation, forming reverse current, irreversibly destroying the function of CL, MEA or bipolar plate [115].

Since the normal operating temperature of the fuel cell is about 80 °C, the generated water exists in the form of gas and liquid mixture and is discharged from MEA by excess cathode reaction gas (air or oxygen) through capillary force of CL and GDL [116]. The reaction gas is discharged from the stack in time through the flow channel in the bipolar plate. The discharge principle of the anode water is the same as that of the cathode one. If the drainage is not timely, it is easy to cause CL flooding.

In addition, water flooding not only occurs in CL and GDL but also in the gas flow field of the bipolar plate, which will seriously affect the distribution and transportation of reaction gas, lead to the shortage of local fuel supply and affect the performance of fuel cell [117,118].

#### 2.5.4. Degradation of CL

Durability is an important research direction in regard to fuel cells and is an important index for their commercialization. The CL degradation is one of the main factors of fuel cell performance degradation. Through the study of the degradation mechanism of CL materials, the influence of various factors on the degradation of CL materials can be fundamentally understood, which provides a theoretical basis for improvement the durability of MEA and fuel cell stack. In this section, the influence mechanism of degradation of ionomer, carbon support and catalyst on MEA performance will be further reviewed.

##### Degradation of Ionomer

Ionomers exist in both PEM and CL, and their degradation directly affects the structure and properties of PEM and CL [119]. During the operation of fuel cells, H_2_O_2_ (two electron pathway) may be generated, which reacts with metal divalent ions to form HO· and HOO· radicals. These radicals directly destroy the structure of perfluorosulfonic acid (PFSA) and react with the hydroxyl groups at the end of polymer chain to generate HF and CO_2_, thereby destroying the function and integrity of Nafion ionomer [120,121,122,123,124].

Because of the different position and working environment of ionomers in CL and PEM, their degradation mechanism is also different. Compared with PEM, the ionomer in CL is closer to the active site. The advantage of being close to the active site is that Pt particles can remove some harmful substances that attack the ionomer, which is beneficial to alleviate the ionomer’s degradation. The disadvantage is that there will be more intermediates and water around the reaction interface, which will aggravate the degradation rate of the ionomer. In general, it is not clear which factor has more influence on the degradation rate. In addition, it is still difficult to distinguish ionomer from Pt/C by traditional characterization methods, and it is uncertain whether F^−^ comes from CL or PEM. More advanced testing and characterization methods are needed to study the degradation technology of the ionomer. For example, Zhang et al. [125] used XPS to monitor the concentration of CF_3_ and CF_2_ in CL of a fuel cell before and after 300 h operation at high current density. The experimental results show that the fluorine concentration on the CL surface decreases from 50.1 to 38.9%, which is consistent with the decrease of CF_3_ and CF_2_. This indicates that Nafion in CL is degraded after 300 h operation.

##### Corrosion of Carbon Support

Carbon support and its interconnection form a carbon binding network, which provides a channel for electron transport. The porosity and pore size distribution, hydrophobicity and electrical conductivity of carbon materials together affect the gas and electron transport and discharge capacity of CL and GDL in the cell. If the carbon material is corroded, the electron transport and discharge capacity of MEA will be weakened, and the CL pore structure will partially collapse in severe cases. Carbon materials are unstable under the conditions of high temperature, high oxygen concentration, insufficient fuel and high potential and easily oxidize into CO and CO_2_ [126,127].

$$C+2H_{2}O\to {\mathrm{CO}}_{2}+4H^{+}+4e^{-}\;E0=0.207\;V\;\mathrm{vs}.\;\mathrm{RHE}$$

$$C+H_{2}O\to \mathrm{CO}+2H^{+}+2e^{-}\;E0=0.518\;V\;\mathrm{vs}.\;\mathrm{RHE}$$

$$\mathrm{CO}+H_{2}O\to {\mathrm{CO}}_{2}+2H^{+}+2e^{-}\;E0=-0.103\;V\;\mathrm{vs}.\;\mathrm{RHE}$$

**Figure 7:**
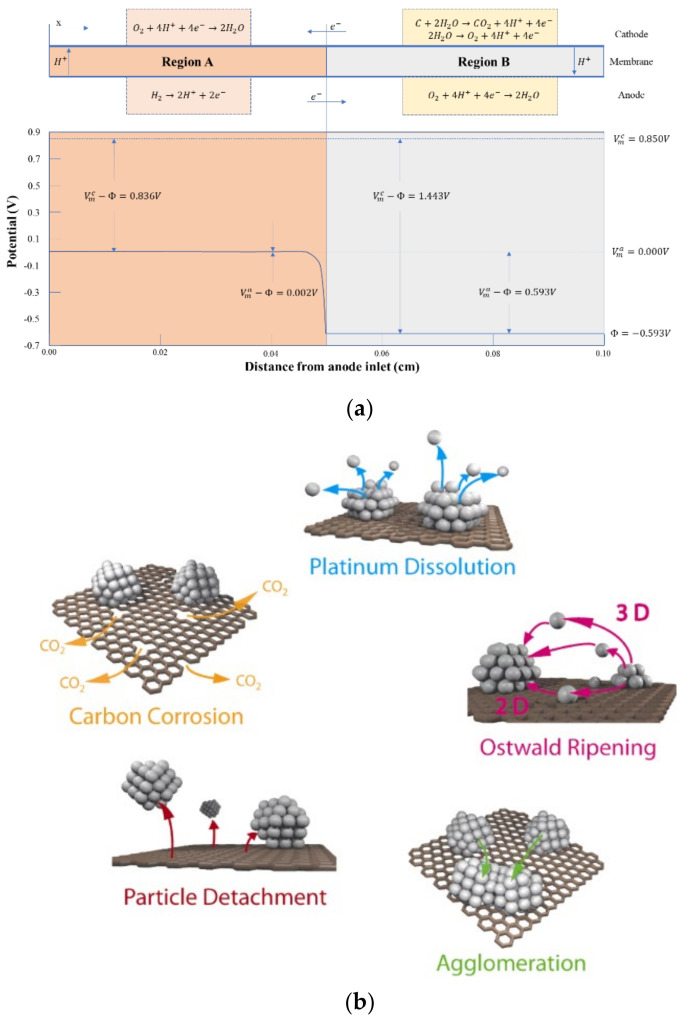
(**a**) Mechanism of reverse current corrosion of carbon support [128]. Copyright 2005 ECS transactions. (**b**) Schematic diagram of degradation mechanism of Pt/C catalyst [129]. Copyright 2014 Beilstein J. Nanotechnol.

Theoretically, carbon will corrode at a potential above 0.207 V, but in practice the corrosion is more pronounced at a potential above 1.2 V. During fuel cell operation, corrosion can be divided into two situations: overall fuel shortage and local fuel shortage [124]. Overall fuel shortage refers to the negative voltage caused by the overall lack of hydrogen in single or multiple cells during the operation of stack, which lowers the cathode potential and causes carbon corrosion. Local fuel shortage refers to the generation of hydrogen/air interface during the start-up or stop of stack, which will lead to the huge difference of electrode potential. There is also a large potential difference in the corresponding cathode region, which leads to carbon corrosion and oxygen precipitation. This phenomenon is called “reverse current corrosion mechanism”, as shown in Figure 7a.

Before the start-up of the stack, the inlet and outlet are always exposed to the air; the cathode and anode are filled with air, and the electrode potential of the cathode and anode is close to the thermodynamic reversible potential of ORR (1.23 V), so the cell voltage is 0 V. When hydrogen flows into the anode but is not fully charged, the cell is divided into an upstream hydrogen rich zone (Zone A in Figure 7a) and a downstream hydrogen deficient zone (zone B in Figure 7a). *V_m_^a^* refers to the anode metal potential; Φ refers to the electrolyte potential, and *V_m_^c^* refers to the cathode metal potential. In the hydrogen rich region, due to the introduction of hydrogen, HOR occurs at the anode side and ORR occurs at the cathode side, and the cell voltage rises to about 0.85 V. Because HOR responds very fast, the anode electron potential is close to the equilibrium potential of HOR (*V_m_^a^* = 0 V, Φ = −0.002 V). Due to the high conductivity of the solid phase, the solid phase potentials of cathode and anode change little along the direction of hydrogen inflow, so it can be considered that in the downstream hydrogen deficient region, *V_m_^a^* and *V_m_^c^* are still 0 V and 0.85 V. But different from the upstream, the downstream anode is surrounded by oxygen, so the electrode potential should be close to the theoretical reversible potential of ORR. Because *V_m_^a^* is a constant of 0 V, the potential of electrode will drop to a negative value in the downstream region B. Reiser et al. [128] found by calculation that the value is about −0.593 V vs. RHE, while the cathode side solid-state potential in this region is about 0.85 V, so the cathode electrode potential is as high as about 1.45 V, which leads to a significant increase in carbon corrosion rate. Tang et al. [130] found that, after repeatedly starting and stopping of fuel cell stack, the hydrogen/air interface formed on the anode side would lead to the rapid decay of cathode electrode, and the cathode CL thickness, ECSA and performance were significantly reduced. A double cell structure was used to measure the cathode potential up to twice the open circuit voltage (OCV), and the external corrosion current between the two cells was detected and quantified to verify the reverse current corrosion mechanism.

##### Degradation of Catalyst

In order to reduce the high surface free energy, Pt nanoparticles usually migrate and agglomerate or dissolve and deposit into larger particles, which will lead to catalyst failure. In addition, Pt particles are very sensitive to external pollutants, such as a small amount of carbon monoxide [131], sulfide [132], nitrogen oxide [133] and metal ion pollutants during fuel cell operation, which will lead to Pt particles “poisoning” and inactivation [134]. The main degradation mechanism of Pt nanoparticles is shown in Figure 7b.

(1) Dissolution and redeposition of Pt particles [135] (Ostwald ripening): Within the fuel cell environment, Pt nanoparticles tend to dissolve into ionic form at high potential and re-precipitate on the surface of large particles at low potential, resulting in the continuous reduction of small particles and the continuous growth of large particles, or the dissolved Pt ions will diffuse into PEM and be reduced to Pt particles in the presence of hydrogen. The dissolution process of Pt particles can be described as follows:
  $$\mathrm{Pt}\;\mathrm{particles}\;\mathrm{dissolve}:\;\mathrm{Pt}\to {\mathrm{Pt}}^{2+}+2e^{-};$$
  $$\mathrm{Formation}\;\mathrm{of}\;\mathrm{Pt}\;\mathrm{oxides}:\;\mathrm{Pt}+H_{2}O\to \mathrm{PtO}+2H^{+}+2e^{-};$$
  $$\mathrm{Pt}\;\mathrm{oxide}\;\mathrm{dissolved}:\;\mathrm{PtO}+2H^{+}\to {\mathrm{Pt}}^{2+}+H_{2}O$$

Darling et al. [136] considered that the dissolution of Pt particles is very slow at low potential, which can be ignored. At high potential, Pt oxide is easy to form, but the dissolution reaction rate of PtO is slow, which inhibits the dissolution of Pt particles. Generally, Pt particles dissolve easily between 0.8~1.1 V. The dissolved Pt ion may replace H^+^ on the side chain sulfonic group of Nafion, which will reduce the number of sulfonic groups available for H^+^ exchange in PEM or CL, resulting in the decrease of proton conduction capacity of MEA. Almost all cations have a higher affinity for sulfonic acid groups than H^+^ [137].

(2) Migration and agglomeration of Pt particles: In order to reduce the higher surface energy caused by the smaller particle size, Pt nanoparticles usually migrate to agglomerate or dissolve and re-deposit into larger particles, thus reducing ECSA and reducing the catalytic performance [138,139].
(3) Corrosion of carbon support [130,140]: It is consistent with the degradation mechanism of carbon materials in CL described in Section Corrosion of Carbon Support. Under high potential conditions, the carbon support of catalyst is oxidizes and corrodes easily, resulting in the loss of catalytic activity of Pt nanoparticles due to the lack of electron transport channel. At the same time, the electrons released from the carbon corrosion reaction will further accelerate the dissolution and precipitation of Pt particles.

**Figure 8:**
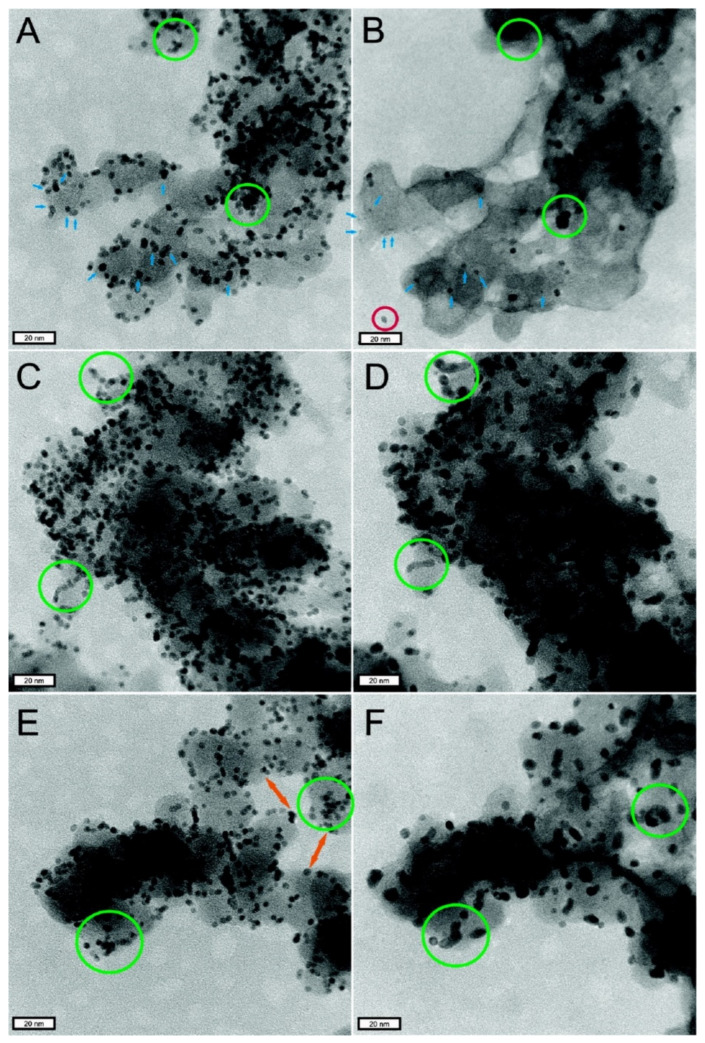
ILTEM images at initial (**A**,**C**,**E**) and after 3600 cycles (**B**,**D**,**F**). Green circles indicate aggregates, red circles indicate desorption Pt particles, blue arrows indicate smaller Pt particles due to dissolution, and orange arrows indicate changes in support structure [141]. Copyright © 2012 American Chemical Society.

Meier et al. [141] used Identical Location Transmission Electron Microscopy (ILTEM) to observe the failure process of Pt catalyst, as shown in Figure 8, which can clearly observe the aggregation, separation, and dissolution of Pt particles as well as the corrosion of carbon support.

In addition, the size of the Pt-based nanoparticle catalyst has a great relationship with its stability [142]. Of course, in terms of the activity of the Pt-based catalyst, the smaller the Pt particle size, the more active sites that can be used for ORR are exposed, the larger the electrochemical active area, and the higher the corresponding catalytic activity. However, smaller particles do not always mean better performance. When the diameter of Pt nanoparticles is reduced to about 1 nm, the interaction between the particle surface and oxygen or oxygen-containing intermediates (-OH, -OOH, etc.) is strong, which affects their dissociation and adsorption but reduces the reaction rate of ORR. In terms of the decay of Pt nanoparticles, due to the influence of the Gibbs–Thompson effect, compared with the larger Pt nanoparticles, the smaller Pt nanoparticles have smaller cohesion and are more easily dissolved, leading to the Ostwald ripening effect, that is, the larger Pt particles continue to grow, while the smaller Pt particles continue to shrink until they disappear, and thus, the ORR activity of the catalyst decreased [143]. It is generally believed that a catalyst particle diameter between 2 and 4 nm has better ORR performance and stability [144].

### 2.6. Structural Optimization of CL

According to the reaction mechanism of the fuel cell, the reaction gas permeates from GDL to the active site, and it must pass through the pore structure of SL, MPL and CL. Therefore, the concentration of reaction gas (hydrogen or oxygen) is the highest near the interface of bipolar plate|SL and the lowest near the interface of PEM|CL. For ORR, the concentration of proton transferred from PEM is the highest on the inner side of CL (PEM|CL interface) and gradually decreases along CL (from PEM to CL). Therefore, the homogeneous phase is not the optimal CL structure. Theoretically, the MEA performance can be optimized by CL gradient design. Parameters such as Pt loading, ionomer content, porosity and others can be used as the basis of CL gradient design optimization. This section mainly discusses the CL performance optimization from the perspective of structural design.

For CL, higher reactivity, lower proton and electron transfer resistance, faster and uniform reaction gas penetration rate and water balance ability, and minimum Pt loading are the goals of CL design optimization. We know that proton conduction in CL is realized by ionomer. The electron transfer starts from the surface of Pt particles and is realized through the carbon binding network, which is related to the properties of carbon materials used and the pore structure of carbon binding network in CL. Therefore, the first idea of high-performance CL design is to change the properties and structure of the material itself, so as to realize the orderly transportation of protons, electrons, reactant gas and product water in CL.

Qi et al. [145] used a new proton conductor poly-pyrrole/sulfonated polystyrene to prepare the electrode. The poly-pyrrole/sulfonated polystyrene has both proton and electron conduction functions, which overcomes the disadvantage of low catalyst utilization due to Nafion excessive coverage Pt and carbon support. Ye et al. [146] use carbonized microporous polyacrylonitrile foam as Pt support; the specific surface is 1000~20,000 m^2^ g^−1^, and the pore size is 1–10 μm. High dispersion of Pt particles was achieved. The electrode adopts 40 μm thick GDL, which reduces the gas diffusion resistance and the electronic resistance, accelerates the discharge of product water, and has better power generation performance.

Chen’s team [147] prepared ordered nanostructured MEA based on independent Pt/VACNTS/Nafion membrane/Pt/VACNTS. The ECSA of MEA with Pt/VACNTS/Nafion membrane/Pt/VACNTS was 78.72 m^2^ g_Pt_^−1^, which was significantly higher than that of conventional MEA (52.22 m^2^ g_Pt_^−1^). Meanwhile, the MEA mass specific power density of ordered nanostructures in H_2_/O_2_ PEMFC (0.1 MPa, 80 °C) was 1.43 kW g_Pt_^−1^, which was 50% higher than that of conventional materials (0.70 kW g_Pt_^−1^). Zeng et al. [148] developed an ordered structure PEMFC based on vertically aligned PtCo bimetallic nanotube arrays. Adding appropriate proportion of pore forming agent can improve the pore structure of CL, form gas transmission channel, and improve the MEA gas transmission capacity [149,150].

On the other hand, considering the continuity of electrochemical reaction, continuous reactant inputs and product outputs are required to ensure the continuous reaction of active sites [151]. However, due to the gradual consumption of reactants, the reactions in CL are not uniformly distributed in space but have a certain gradient difference. From this point of view, CL gradient is another way to optimize MEA performance. For example, the CL pore gradient structure is used to optimize the internal water and gas transportation. The CL Pt loading gradient structure was used to optimize the Pt loading and improve the utilization rate. The CL Nafion content gradient structure was used to improve the proton transfer. At present, the CL gradient structure is mainly realized by building a multi-layer structure.

Su et al. [152] prepared CL with a Pt loading gradient structure by using a double CL structure design. On the inner side of the membrane, a high Pt loading CL is used to concentrate Pt particles on the side close to PEM. In order to maintain the CL thickness, a low Pt supported CL is used in the outer layer. The test results show that, the current density of the dual CL structure is 35.9% higher than that of the conventional homogeneous CL structure at 0.6 V. Taylor et al. [153] prepared a three-layer CL structure with Pt/C ratio gradient by spraying method: starting from the PEM side, the Pt loading decreased in turn (50/20/10%). Compared with the uniformly distributed catalyst (20%), the catalyst with gradient distribution has better performance, but the total Pt loading is almost the same.

Jain et al. [154] studied in detail the effect of an ionomer gradient in CL on fuel cell performance. They prepared three different types of CL: (1) Homogeneous CL with 30% Nafion content. (2) From surface to interior (from carbon paper to PEM), the Nafion content in CL decreased gradually (40~30~20% for three layers). (3) The Nafion content in CL increased gradually from outside to inside (20~30~40% for three layers). The CL proton conductivity and electrochemical properties were studied by physical (porous distribution) and electrochemical (cyclic voltammetry (CV) and EIS). The results showed that when the Nafion content increases gradually from the outside to the inside (from carbon paper to PEM), that is, when the Nafion content is higher on the PEM side and lower on the carbon paper side, the performance is higher than that of the cell assembled with uniform distribution of Nafion. Kim et al. [155] constructed a double CL, studied the influence of Nafion content in CL on the performance, and verified the experimental results of Jain P et al. Xie et al. [156] verified through experiments that, in the case of medium and high current density, compared with the CL with uniform Nafion distribution (30%), the Nafion content gradually decreases from PEM to GDL side (40~30~20%) and can maximize the cell power.

Qiu et al. [157] designed and prepared cathode CL structures with different porosity and Pt loading, which had higher Pt content in the inner layer and higher porosity in the outer layer. Under the conditions of Pt loading of 0.28 mg cm^−2^ and hydrogen/air test, the peak power reaches 0.76 W cm^−2^. It is concluded that the higher Pt content in the inner layer provides more catalytic active sites, and the higher porosity in the outer layer alleviates the risk of electrode flooding. This design not only improves the Pt particles utilization rate in CL but also reduces the mass transfer resistance. Ye et al. [158], using commercial Pt/C catalyst as active component, prepared a double-layer CL with reverse gradient distribution of Pt loading and porosity through vacuum filtration. The single cell test results show that the performance of the proposed method is 11% higher than that of the traditional method.

At present, the nanostructured thin film (NSTF) developed by the 3M company has the best performance in the ordered CL [159]. In addition, there are also many studies on the preparation of self-humidifying CL to optimize its power generation performance [160,161,162,163].

## 3. Preparation of CL

In the early stage, the CL is mainly based on PEM; the mixture of pure Pt and Teflon particles is used as catalyst, which is hot-pressed on the membrane, and the Pt loading is of up to 10 mg cm^−2^. As a scarce precious metal, Pt has become an important factor limiting the large-scale application of membrane electrodes.

In order to improve the utilization of Pt, the Pt support on carbon particles was used to make the catalyst slurry, and PTFE was used as the binder in the later period. The two above materials are mixed uniformly at a certain ratio to prepare CL on GDL. After that, it is sintered, impregnated with Nafion solution, and resintered; then, the membrane electrode is formed by membrane hot-pressing. This preparation method is the most traditional MEA preparation method-GDE method [164,165]. The Pt loading is reduced to 4 mg cm^−2^, but the utilization rate of Pt still very low.

In the middle and late 1980s, fabrication techniques of GDE continued to improve. The Los Alamos National Laboratory (LANL) in the US proposed to use Nafion instead of PTFE to impregnate Pt/C to prepare porous GDE and then press it on the membrane. Nafion improves the proton transfer conductivity, so that the Pt loading is reduced from 4 mg cm^−2^ to 0.4 mg cm^−2^ but still maintains high catalytic activity [12,25,166]. This breakthrough makes possible the commercialization of MEA. Lee et al. [73] studied the effect of Nafion impregnation on the polarization characteristics of the electrode. Experiment results show that, when the amount of Nafion impregnation is 1.9 mg cm^−2^, the electrode performance is the best. Compared with non-impregnated Nafion, the charge transfers resistance decreases by 1.463 Ω cm^2^, and the initial potential E_0_ increases by 23 mV (2 atm, 70 °C, H_2_/O_2_, 0.4 mg cm^−2^ Pt loading). When the immersion amount is higher, the active area increases with the increase of Nafion loading, but the electrode resistance increases due to the increase of membrane thickness and the diffusion resistance of Nafion to CL, thus hindering the mass transmission. In 1992, LANL improved the method of MEA preparation and further reduced the Pt loading to 0.13 mg cm^−2^. In 1995, the Indian Science Foundation Electrochemical and Energy Research Center (CEER) prepared MEA with Pt loading of 0.1 mg cm^−2^ by spray dipping method, and its performance is comparable to Pt loading of 0.4 mg cm^−2^ [167].

The U.S. DOE’s technical target values for the total Pt group metal content, total loading and mass activity of fuel cell catalysts in 2020 are 0.125 g kW^−1^@150 kPa, 0.125 mg cm^−2^ and 0.44 A mg_Pt_^−1^@900 mV_IR-free_ [168]. After several generations of technological innovation, the preparation technology gradually makes this goal possible. At present, the application of low Pt loading catalyst is still mainly focused on the experimental research, especially the application of multi-component catalyst and the ordered structure of MEA, which reduces the Pt loading to 0.1 mg cm^−2^ or even lower; however, the Pt loading of commercial catalyst is about 0.4 mg cm^−2^, so the road to large-scale production is still difficult.

### 3.1. Coating Process of CL

**Figure 9:**
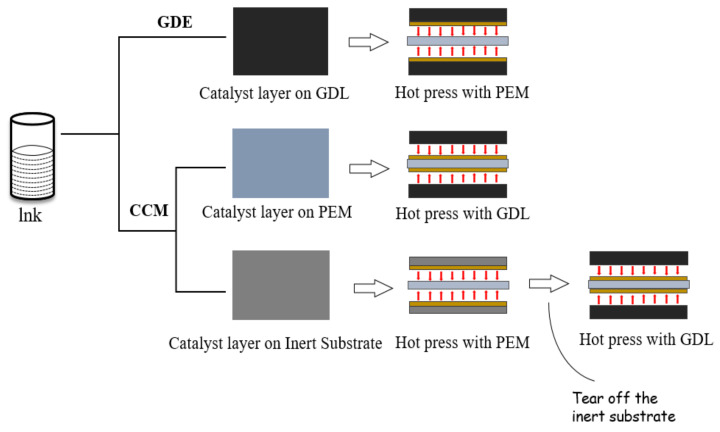
Schematic diagram of CL preparation process [169]. Copyright 2018 Elsevier.

The CL preparation process affects the distribution of ionomer and carbon binding network and the formation of the porous structure. As shown in Figure 9 [169], GDE is the first generation of the CL coating process, which is widely used in the early stage of the fuel cell. CCM is the second generation of the CL coating process, which is the mainstream process at present. The principle of CCM is that the catalyst slurry is directly coated on PEM, and the porous structure is formed after drying, which can realize the transfer of electrons, the transport of reaction gas and the discharge of product water. Compared with the GDE process, the CCM process can significantly reduce the CL|PEM interface contact resistance [170]. In addition to direct coating onto PEM, CCM can also be realized by transfer printing [171,172]. The catalyst slurry was coated on inert substrates such as PET film, dried and then transferred to PEM by hot pressing. PEM with CL is formed after peeling the PET film, which can avoid the membrane expansion problem caused by direct coating of wet slurry on PEM [173]. Zhang et al. [174] compared MEA prepared by the GDE process (Pt loading 4 mg cm^−2^) and the CCM process (anode Pt loading 0.4 mg cm^−2^, cathode Pt loading 0.8 mg cm^−2^). In the low current density region mainly affected by activation, there was no significant performance difference between the two processes. However, with the increase of current density to the ohmic polarization region, the performance of the CCM process is better than that of the GDE process due to the lower internal resistance. Shahgaldi et al. [169] studied the effects of different preparation processes on MEA performance. The results show that the MEA performance can be improved by coating Nafion on CL by low temperature decal transfer method (LTDT) and GDE. In the CCM process, the addition of a Nafion layer will deteriorate the MEA performance. When the Pt loading is reduced to 0.125 mg cm^−2^ (the Pt loading decreased by 75%), the cell performance of GDE decreases significantly (about 75%), while the cell performance of the other two processes (LTDT and CCM) decreases by less than 30%. However, no matter which coating process is used, its realization forms are varied. Here are two main coating preparation methods.

(1) Spraying method [175]

Spray method is to spray catalyst slurry directly on GDL or PEM by spraying equipment and forming CL after drying. In order to ensure the uniformity and consistency of CL and the limitation of spraying equipment, the solid content of catalyst slurry is generally low (wet) and requires a small amount of continuous spraying for many times, which seriously limits the efficiency of the spraying method. At the same time, during the spraying process, it is necessary to heat the substrate (in the case of PEM) to make the dispersed components (such as water or organic solvent) evaporate rapidly, so as to prevent the secondary interference caused by repeated spraying. The CL prepared by the spraying method is thinner, the slurry component is uniformly distributed, and the CL has good repeatability and stability [176]. Due to being time-consuming and to its low efficiency, the spraying method is difficult to achieve large-scale production in practical application, but it is a common method for small MEA sample preparation in laboratory [173,177].

(2) Slot die coating [178,179]

The slot coating machine is a widely used piece of coating equipment in CL preparation. The precision injection pump is used to control the feeding speed (feeding quantity), and the coating thickness is controlled by the height between the mold and the substrate, the gap size of the mold head, the substrate moving speed and other factors. The coating speed is controlled by the relative speed between the die head and the substrate. According to the principle of slot die coating, single-piece coating, roll-to-roll coating and intermittent coating are derived. As the name suggests, the single-piece coating is to cut the substrate into the ideal size and then coat the slurry one by one. The advantage is that coating parameters can be controlled one by one, which is suitable for differential and small batch MEA production. The disadvantages are high labor cost, long time-consuming, the head and tail of the single piece are prone to lack of slurry, accumulation of slurry, poor repeatability, more slurry loss, and poor economic benefits. Roll-to-roll coating is the main way of MEA mass production. It achieves continuous coating operation on the entire substrate roll, similar to the roll-to-roll production line of lithium-ion electrode. The advantages are automatic operation of the coating process, less human intervention, good CL consistency and high coating efficiency. The disadvantages are the high precision equipment requirement, large one-time investment, cumbersome debugging process in the early stage, and the subsequent cutting process will also cause slurry waste. The roll-to-roll coating method is suitable for large-scale, high consistency continuous production. Intermittent coating is a kind of coating method similar to roll-to-roll, the only difference is that there is a fixed gap between each coating unit according to the requirements, which is conducive to the subsequent electrode cutting process and reduces the waste of slurry.

Other coating methods include brush coating method [180,181], dry powder spraying method (mixing and grinding CL components into particles, directly spraying on the surface of PEM) [182,183], screen printing method [184], sputtering coating (ion bombarding target, atomic deposition on the surface of substrate to form a thin film) [185], electrochemical deposition (using electrochemical methods to deposit Pt at a specific location) [186,187,188], etc.

The coating efficiency and effect of CL are closely related to the characteristics of the slurry used. The slurry formula, dispersion method and coating process directly affect the structure and performance of CL. The surface tension, viscosity, viscoelasticity, solid content, solvent evaporation rate and other parameters of the slurry should adapt to the coating process. The coating process should be able to realize simple and accurate control. Taking the slot die coating process as an example, when preparing cathode CL, the efficiency of the coating process should be improved from multiple coating to one coating, accurately control the edges and corners around the coating range, head and tail, avoid material accumulation at the head and tail, and ensure the consistency of coating thickness. Only in this way can the dried CL have good thickness consistency, ensure the porous structure of CL, form as many effective three-phase interfaces as possible, and improve the utilization rate of the catalyst.

### 3.2. Drying Process of CL

The CL drying refers to the process in which the slurry coats on the substrate (taking PEM as an example), the substances irrelevant to the CL components (such an alcohol dispersant, pore forming agent, water, etc.) evaporate from the liquid phase wet coating, and the solid phase dry coating is formed. After drying, the CL’s composition and distribution, porosity, hydrophilic and hydrophobic characteristics have been basically set, so the CL drying process has a direct relationship with the performance, lifetime and other indicators of MEA.

**Figure 10:**
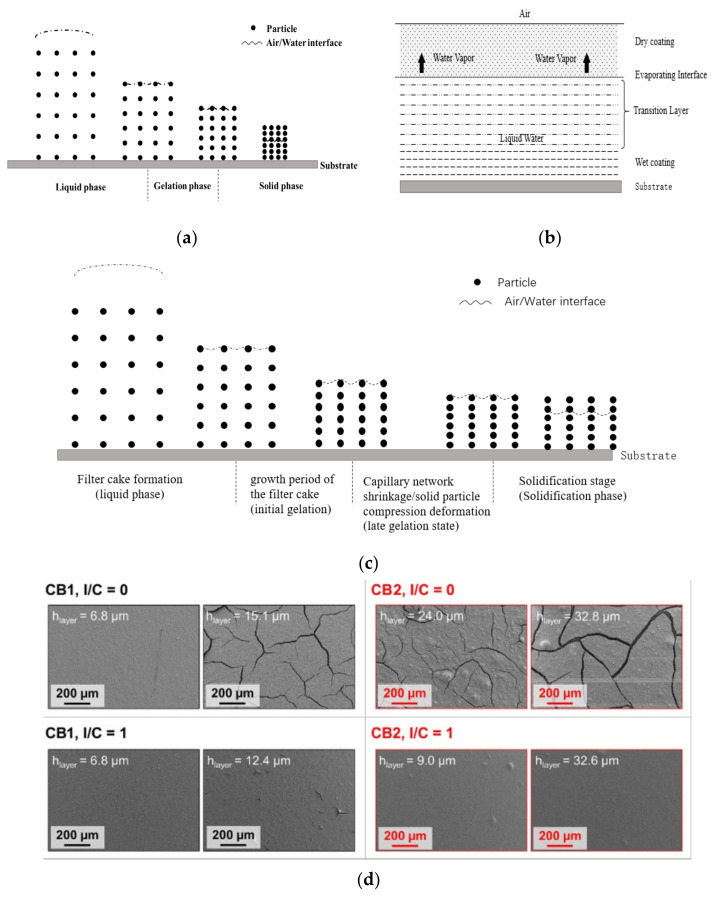
(**a**) Classic consolidation mechanism of coating layer proposed by Lepoutre [193]. (**b**) Croll’s film formation model [194]. (**c**) Schematic of the consolidation mechanism of filter cake layer on coating surface [191]; (**a**–**c**) adapted from [195]. (**d**) SEM micrographs of CL cracks after drying [27]. Copyright 2019 Elsevier.

Based on the classical Lepoutre’s solidification theory [189,190,191,192,193] (Figure 10a), combined with the Croll film forming theory of latex [194] (Figure 10b), Zang et al. [191,193,195] carried out research on a coating solidification mechanism related to coated paper in the paper making industry (Figure 10c).

The classic theoretical mechanism of coating solidification of Lepoutre is shown in Figure 10a [193]. An important boundary condition of this model assumes that the slurry coating on the substrate still maintains uniform distribution in the direction perpendicular to the substrate. By analogy, the drying process of wet CL can also be divided into three stages: liquid phase, gelation phase and solid phase.

(1) After the catalyst slurry coats on PEM, the wet coating surface is covered by a water-like film. The coating has the characteristics of stable suspension, such as particles being able to move under the action of thermal movement and diffusion of liquid molecules, and the wet coating can flow under the action of external forces. Liquid evaporation mainly occurs at the interface between the wet coating surface and the air. The volume of the evaporating solvent is equivalent to the change of volume in the wet coating. This process is called the liquid phase.
(2) The duration of the liquid phase relates to many factors, such as the slurry solid content, the temperature, humidity and so on. As the solvent continues to evaporate, the water-like film on the wet coating surface disappears. A three-dimensional capillary network forms between the carbon-based Pt catalyst and the Nafion binder within the coating, which could still contract and deform although it can greatly restrict the free movement of particles. In this process, the evaporation of solvent still occurs on the coating surface, but it is different from the position of the liquid phase, at the interface between the meniscus surface formed by the solvent in the capillary and the air. Since CL has formed the capillary structure, the solvent can be transferred to the evaporation surface (the interface between the meniscus surface of the capillary and the air) by capillary force to maintain the solvent evaporation process. Due to the continuous evaporation, the volume of the coating further shrinks and the gap between the solid particles (catalyst particles and Nafion particles) reduces gradually, resulting in gradual increase of capillary force. When the solid content reaches a certain degree, the solid particles compress and deform under the action of capillarity until they no longer change. This process is called the gelation phase.
(3) Finally, there is the solid phase. When the solid particles of the coating are no longer deformed, the amount of solvent in the coating is insufficient to continue to transfer to the coating surface through the capillary tube for evaporation, and the evaporation interface reduces from the outside to the inside gradually, causing air to occupy the void above the evaporation interface. This process determines the spatial distribution and the pore size distribution of the catalyst, Nafion and other compositions within CL.

According to the theory of Croll latex film forming (Figure 10b), during the drying process, the formation of the dry coating is due to the particles being close to each other, deforming and bonding on the surface. With the evaporation of solvent on the coating, the evaporation interface deepens from the outside to the inside gradually, and the coating interface thickens from the outside to the inside gradually, until the wet coating disappears completely.

On the basis of the above two theories, Zang et al. [191] put forward the four-stage theory of evaporation, that is, the solidification mechanism of filter cake layer formed on the coating surface. The schematic diagram of the mechanism is shown in Figure 10c, and it can be divided into four stages:

(1) Filter cake formation (liquid phase). The stage from the slurry coats on substrate, to continuous water-like film disappears on the wet coating surface.
(2) The growth period of the filter cake (initial gelation). The stage from the continuous water-like film disappears, and the free water disappears in the wet coating. Free water does not contain the water confined to capillaries formed by particles.
(3) Capillary network shrinkage/solid particle compression deformation (late gelation state). This stage begins with the evaporation of water in the capillaries, until the coating is no longer in compressed deformation. In the second stage, the loss of free water and the shrinking of coating volume lead to the decrease of particle spacing, and the increase of capillary force. The evaporation interface stays on the meniscus surface of the coating surface. With the increase of capillary force, the compressible particles in the coating deform gradually until the particle spacing cannot be reduced. This stage relates to the content of compressible material and its elastic modulus within the coating.
(4) Solidification stage (solidification phase). In the final stage, the coating structure has been formed and no longer is compressed or deformed with the increase of capillary force. The evaporation interface moves down gradually until it makes contact with the substrate, which is the same as the last stage of Lepoutre’s classic coating solidification theory.

Therefore, from the mechanism analysis, during the CL preparation process, the appropriate control of the drying intensity can prevent the cracking caused by the imbalance of capillary force inside the CL (Figure 10d).

Talukdar et al. [192] proved that, during the fabrication of MEA, the drying process directly affects the CL micropore structure and then affects the utilization of the precious metal, such as Pt, thus affecting the performance of the fuel cell. They innovated the way of freeze-drying the electrode, using the sublimation principle to remove the solvent in the wet coating, in contrast to the traditional drying technology using an oven, and made the porosity 3.5 times and ECSA 1.5 times.

## 4. Conclusions and Prospects

### 4.1. Conclusions

MEA is the core component of the fuel cell, which has an important influence on its performance, cost and durability. CL is the key part of electrochemical reaction in MEA, and understanding the composition and function of CL, preparation process and influencing factors of degradation is the basis for further study and improvement of MEA performance and durability.

This paper introduces the CL material composition and function; discusses the two key interfaces with CL: PEM|CL and CL|MPL; introduces the proton, electron and mass transport and the degradation mechanism of CL and its preparation and optimization, especially the coating process and drying process; and expounds the CL failure and the optimization of high-performance CL.

### 4.2. Prospects and Challenges

Low-Pt or non-Pt catalysts are the development direction of catalysts in the future, but most of them are still at the level of laboratory research and have few commercial applications. The research on the consistency control and key factors in CL preparation process is not deep enough in areas such as how to avoid coating stripes and defects. The binding mechanism between Nafion and catalyst particles and the effect of solvent properties on the binding process need to be further studied. The research of the slurry preservation method needs to be optimized on how to maintain its consistency and stability. In terms of how to improve the preparation efficiency of CL, continuous improvement is also needed.

After long-term operation of the fuel cell, the delamination of CL from PEM and MPL interface and the thinning of CL and PEM need to be focused on. The influence and mechanism of the cracks on the performance and durability of CL are not clear and need to be further explored. The decay mechanism of ionomer within CL and its influence process on the performance and durability of CL are not clear enough.

Finally, since the CL durability test generally takes a long time, it is also an important direction to establish the model and standard of the accelerated aging test and explore the corresponding relationship with the actual durability test.

## Data Availability

Not applicable.
